# Refined risk stratification helps guiding transplantation choice in adult *BCR::ABL1*-positive acute lymphoblastic leukemia

**DOI:** 10.1038/s41408-024-01055-1

**Published:** 2024-04-24

**Authors:** Cheng Wang, Jianfeng Li, Weiyang Liu, Lingling Zhao, Han Yan, Yuchen Yan, Jiayi Ren, Lijun Peng, Jiaojiao Zhang, Yuanfang Liu, Xiangqin Weng, Yongmei Zhu, Duohui Jing, Jian-Qing Mi, Jin Wang

**Affiliations:** Shanghai Institute of Hematology, State Key Laboratory of Medical Genomics, National Research Centre for Translational Medicine at Shanghai, Ruijin Hospital Affiliated to Shanghai Jiao Tong University School of Medicine, Shanghai, China

**Keywords:** Acute lymphocytic leukaemia, Cancer genomics

Dear Editor,

The integration of tyrosine kinase inhibitors (TKIs) into multiagent chemotherapy regimens has markedly improved the prognosis of *BCR::ABL1*-positive acute lymphoblastic leukemia (*BCR::ABL1* + ALL). Allogeneic hematopoietic stem cell transplantation (allo-HSCT) remains a backbone of contemporary curative treatments for adult *BCR::ABL1* + ALL.

*IKZF1*^plus^ is a genotype characterized by the coexistence of *IKZF1* deletions with deletions in *CDKN2A*, *CDKN2B*, *PAX5*, or PAR1 in the absence of *ERG* deletion [1]. In 2023, the National Comprehensive Cancer Network (NCCN) ALL guideline officially stratified *BCR::ABL1* + ALL into poor-risk [*IKZF1*^plus^ or antecedent chronic myeloid leukemia (CML)] or standard-risk (non-*IKZF1*^plus^ and without antecedent CML) category [2]. Simultaneously, minimal residual disease (MRD) is a well-recognized prognostic factor of ALL. However, there have been no studies on the synergy of the two prognostic factors (*IKZF1*^plus^ genotyping and MRD assessment) in risk stratification. Particularly, although increasing evidence suggests that allo-HSCT may not benefit all patients, the criteria for determining which patients should undergo or be spared from allo-HSCT remain elusive.

In this study, we aimed to refine the risk stratification by integrating *IKZF1*^plus^ genotyping and MRD assessment, thus improving the management of allo-HSCT in *BCR::ABL1* + ALL therapy. Additionally, we preliminarily explored the molecular characteristics of *IKZF1*^plus^ ALL concerning its high-risk phenotype through targeted exome sequencing and RNA sequencing (RNA-seq), providing new insights into mechanistic research.

Here, we analyzed 156 adult patients with newly diagnosed *BCR::ABL1* + ALL in three clinical trials conducted from 5 June 2014 to 15 November 2022. Till 31 December 2023, the overall median follow-up period was 34.6 months (range, 7.5–116.5). The patients were treated with a TKI-based standardized regimen, including imatinib or flumatinib (a second-generation TKI). In order to reduce high-dose chemotherapy-associated toxicity and early deaths, the relatively low intensity of chemotherapy was applied [3, 4]. There was no significant difference in baseline characteristics and survival outcomes between imatinib and flumatinib cohort, allowing a joint prognostic analysis (Supplemental Table 1, Supplemental Fig. 1A–C). In accordance with the Declaration of Helsinki, the study was approved by the Ethics Committee of Ruijin Hospital. [Clinical Trial Registration Number: ChiCTR-ONRC-14004968 [5], ChiCTR2100042248 [6, 7], and ChiCTR2100044308].

A landscape of gene deletions, mutations, and clinical data of the 156 patients was illustrated in Fig. 1A and Supplemental Fig. 2A, B. The patients were classified into *IKZF1*^plus^ group (*n* = 54) or non-*IKZF1*^plus^ group (*n* = 102) [composed of *IKZF1* deletion alone (*n* = 65) and no *IKZF1* deletion (*n* = 37)], according to multiplex ligation-dependent probe amplification (MLPA) results (Fig. 1A). Internal validation (Supplemental Table 4) and whole genome sequencing (Supplemental Fig. 2C) were performed to validate the MLPA technique. Clinical information was provided in Supplemental Tables 2 and 3. The *IKZF1*^plus^ group revealed micro-deletions in *CDKN2A*/*2B* (34.6%), *PAX5* (31.4%), *ERG* (0%), and PAR1 region (0%) gene loci.

**Fig. 1:**
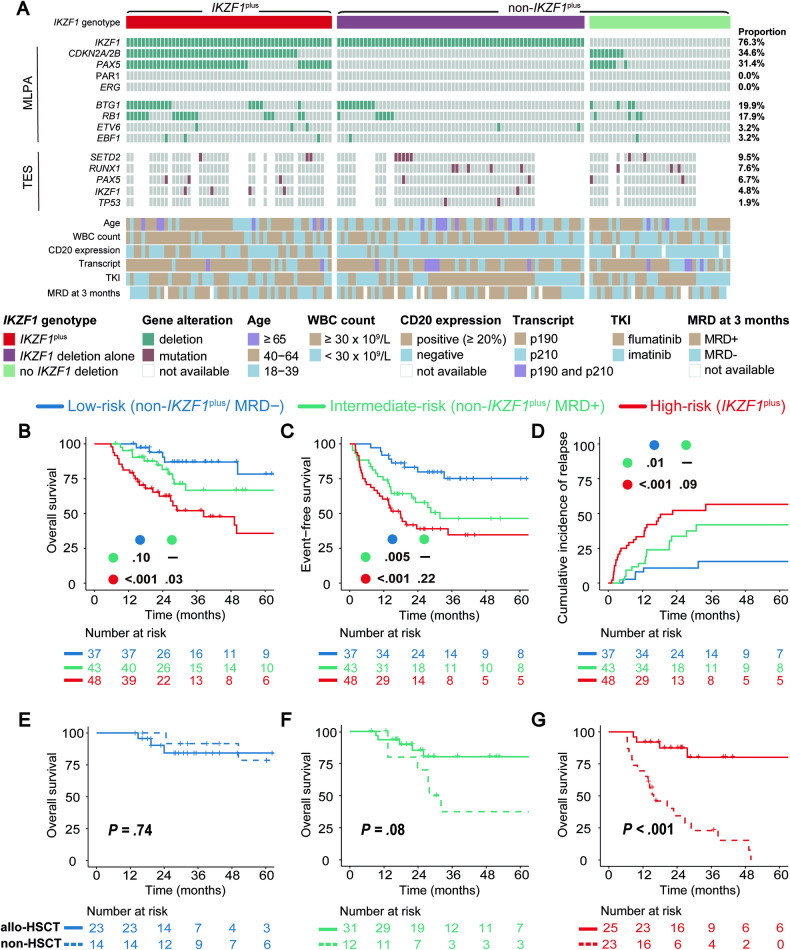
The role of allo-HSCT for three distinct risk groups according to the refined stratification system combining *IKZF1* genotype and MRD status in adult *BCR::ABL1* + ALL. **A** The profile of gene deletions, gene mutations, and clinical characteristics of 156 adult *BCR::ABL1* + ALL patients. Patients are divided into three groups according to *IKZF1* genotypes. Gray columns indicate patients, and rows include three panels: *IKZF1*^plus^ panel (*IKZF1*, *CDKN2A/2B*, *PAX5*, *ERG* gene, and PAR1 region), deletion panel (*BTG1*, *RB1*, *ETV6*, and *EBF1* gene), and mutation panel (*SETD2*, *RUNX1*, *PAX5*, *IKZF1,* and *TP53* gene). Alteration frequencies in each are shown on the right. The last heatmap shows clinical information. **B**–**D** Overall survival, event-free survival, and cumulative incidence of relapse for patients (aged 18–64 years) grouped by revised risk stratification: low-risk (non-*IKZF1*^plus^/MRD−), intermediate-risk (non-*IKZF1*^plus^/MRD+) or high-risk (*IKZF1*^plus^), respectively. **E**–**G** Effects of transplantation on overall survival for three distinct risk groups (aged 18–64 years), respectively. *P* values are calculated using the log-rank test. The number at risk is based on all evaluable patients. Abbreviations: MLPA, multiplex ligation-dependent probe amplification; TES, targeted exome sequencing. *MRD− refers to patients achieving CMR (evaluated by RT-qPCR with a sensitivity of 0.001%) at 3 months of treatment, while MRD+ refers to patients failing to achieve CMR at 3 months in this study.

139 patients (aged 18-64 years) were included in the survival analysis. The OS, EFS, and CIR of *IKZF1*^plus^ group (*n* = 48) were markedly worse than those observed in non-*IKZF1*^plus^ group (*n* = 91) (Supplemental Fig. 3A–C).

MRD evaluation was performed for 125 patients at 3 months of treatment by RT-qPCR (sensitivity: 0.001%). Outcomes of patients who failed to achieve complete molecular remission (CMR) at 3 months (MRD+, *n* = 68) were significantly worse than those with CMR at 3 months (MRD−, *n* = 57) (Supplemental Fig. 3D–F).

Next, incorporating the two risk factors, 125 patients (aged 18–64 years) with available data were stratified into four subgroups [non-*IKZF1*^plus^/MRD− (*n* = 37), non-*IKZF1*^plus^/MRD+ (*n* = 43), *IKZF1*^plus^/MRD− (*n* = 20) and *IKZF1*^plus^/MRD+ (*n* = 25)] (Supplemental Fig. 4A–C). The non-*IKZF1*^plus^/MRD− patients were classified into the low-risk group, demonstrating the best survival outcomes and the lowest relapse rate. In contrast, *IKZF1*^plus^/MRD−, *IKZF1*^plus^/MRD+, and *IKZF1*^plus^/MRD not available (*n* = 3) subgroups had the worst outcomes and highest relapse rates, therefore combined into the high-risk group (*IKZF1*^plus^, *n* = 48), indicating MRD assessment did not contribute to the risk stratification of *IKZF1*^plus^ patients. The remaining non-*IKZF1*^plus^/MRD+ subgroup was stratified as an intermediate-risk group (*n* = 43). Finally, compared to the intermediate-risk or high-risk group, the low-risk group had better OS (*P* = 0.10 or *P* < 0.001), EFS (*P* = 0.005 or *P* < 0.001), and lower CIR (*P* = 0.01 or *P* < 0.001), respectively (Fig. 1B–D, Supplemental Table 5). Overall, the refined risk stratification could better discriminate the prognosis of adult *BCR::ABL1* + ALL patients.

Therefore, based on our refined risk stratification system, 128 patients (aged 18-64 years) eligible for transplantation were analyzed. In low-risk group, OS of allo-HSCT subgroup was similar to non-HSCT subgroup (*P* = 0.74) (Fig. 1E), suggesting that low-risk patients did not benefit from transplantation. Among 23 patients transplanted, three died of infections post allo-HSCT. Among 14 patients without transplantation, one succumbed to heart failure and another due to leukemia progression.

For high-risk patients, OS of allo-HSCT subgroup was significantly higher than non-HSCT subgroup (*P* < 0.001) (Fig. 1G), confirming the necessity of transplantation in high-risk group. Finally, among intermediate-risk patients, OS of allo-HSCT subgroup showed a more favorable trend (*P* = 0.08) (Fig. 1F). Without allo-HSCT, OS of low-risk group was significantly superior to intermediate-risk and high-risk groups, respectively (Supplemental Fig. 5E). While after allo-HSCT, OS of three groups reached a comparable level (*P* = 0.85) (Supplemental Fig. 5F). Overall, our data suggest that allo-HSCT should be spared for low-risk group but is recommended for the other two groups (Supplemental Fig. 6).

Multivariate analysis demonstrated that *IKZF1*^plus^ genotype (all *P* < 0.001) and MRD positivity at 3 months (*P* = 0.01, *P* < 0.001, *P* < 0.001) were independent risk factors for OS, EFS, and RFS, while allo-HSCT (all *P* < 0.001) was an independent protective factor (Supplemental Fig. 7).

Based on RNA-seq data from 137 patients, the developmental stages of all patients were inferenced by using diffusion map-based dimensionality reduction (Supplemental Fig. 8) and lineage signatures (top 100 genes) in Bastian et al. [8]. It is consistent with previous reports, the non-*IKZF1*^plus^ group and *IKZF1*^plus^ group enriched in distinct hematopoietic lineage, including B lymphoid/myeloid lineage, early pro-B cells, and pre-B I/II large stages (Fig. 2A–C) [9, 10]. Using multiparameter flow cytometry [11], similar results at the protein expression level were validated (Fig. 2D).

**Fig. 2:**
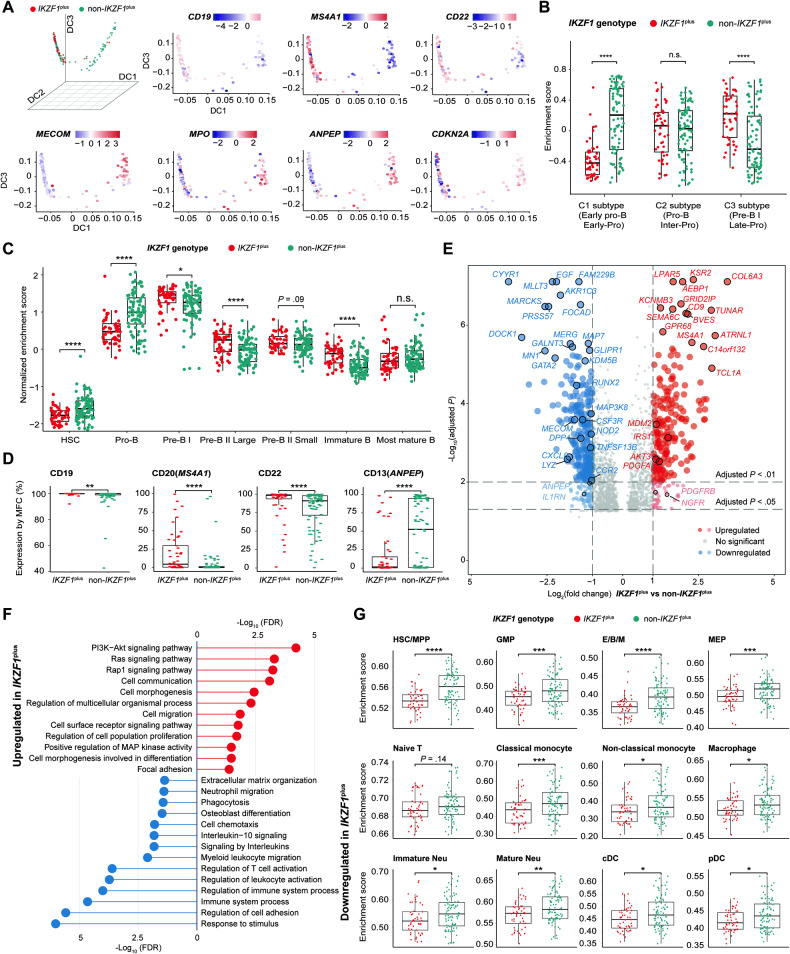
Transcriptome landscape of *IKZF1*^plus^ in adult *BCR::ABL1* + ALL. **A** Diffusion map for visualization of cell lineage differentiation through dimensionality reduction. The top 100 differentially expressed genes (DEGs) between multilineage and lymphoid cluster found by Bastian et al. [8] are subjected to diffusion map analysis, and the first three diffusion components are displayed using a three-dimensional plot. Each point stands for a patient (*n* = 137). Top left panel labels points with *IKZF1*^plus^ (*n* = 49) or non-*IKZF1*^plus^ (*n* = 88) group. Other panels show scaled scores calculated based on single sample gene set enrichment analysis (ssGSEA) method, including B lymphoid lineage markers (*CD19*, *MS4A1*, *CD22*), myeloid lineage markers (*MECOM, MPO*, and *ANPEP*) and *CDKN2A* gene. **B** ssGSEA score between *IKZF1*^plus^ and non-*IKZF1*^plus^ group, using gene signatures associated with three transcriptomic classes that correspond to early pro-B, pro-B and pre-B I differentiation stages in Kim et al. [9]. **C** ssGSEA score between *IKZF1*^plus^ and non-*IKZF1*^plus^ group, using seven B lymphopoiesis stage-specific gene sets identified by Beder et al. [10]. **D** Box plots illustrating expression of B lymphoid and myeloid lineage antigens between *IKZF1*^plus^ and non-*IKZF1*^plus^ group, as measured by multiparameter flow cytometry (*n* = 135, 2 patients without flow cytometry results). **E** Volcano plot shows DEGs between *IKZF1*^plus^ and non-*IKZF1*^plus^ group. Each dot represents one gene. Genes significantly upregulated in *IKZF1*^plus^ are colored in red, and downregulated in *IKZF1*^plus^ are colored in blue. **F** Lollipop chart visualizes enriched pathways or terms that are upregulated and downregulated in *IKZF1*^plus^ group, utilizing Gene Ontology, Kyoto Encyclopedia of Genes and Genomes, and Reactome database. **G** Enrichment score of various cell types between *IKZF1*^plus^ and non-*IKZF1*^plus^ group, using cell type-specific genes from single-cell RNA sequencing data of human normal hematopoiesis cells reported by Zhang et al. [12]. Abbreviations: n.s. no significant, HSC/MPP hematopoietic stem cell/multi-potential progenitor, GMP granulocyte–macrophage progenitor, E/B/M eosinophil/basophil/mast cell progenitor, MEP megakaryocyte and erythroid progenitor, immature Neu immature neutrophil, mature Neu mature neutrophil, cDC conventional dendritic cell, pDC plasmacytoid dendritic cell. *P* values are calculated using Wilcoxon rank-sum test. *P < 0.05, **P < 0.01, ***P < 0.005, and ****P < 0.001.

Several unreported signature genes of *IKZF1*^plus^ group were identified in this work for the first time (Fig. 2E), including upregulated (*TUNAR*, *GPR68*, *TAFA1*, *CD24,* and *TUBB3*) or downregulated genes (*MARCKS*, *CDKN2A,* and *TBL1X*) (Supplemental Table 6). *IKZF1*^plus^ genotype was associated with activation of proliferative pathways [PI3K-Akt and Ras signaling pathways] and inhibition of hematopoietic/immune functions [T cell activation and other immune system process] (Fig. 2F). The above findings then were validated by additional gene sets [12] and immunocyte deconvolution (xCell algorithm) (Fig. 2G, Supplemental Fig. 9A) [13]. Consistently, peripheral blood samples of *IKZF1*^plus^ group showed significantly lower expression of a T cell antigen CD3 (*P* = 0.04) (Supplemental Fig. 9B). Therefore, better immune function may be a positive factor contributing to the improved prognosis of non-*IKZF1*^plus^ group.

This study is the largest cohort to date focusing on *IKZF1*^plus^ aberration and MRD simultaneously in adult *BCR::ABL1* + ALL. Integrating both *IKZF1* genotype and MRD status at 3 months, *BCR::ABL1* + ALL could be further classified into three distinct risk groups: low-risk, intermediate-risk, and high-risk. The refined stratification system not only essentially meets the criterion for 2023 NCCN’s poor-risk category, but also allows for a subdivision of NCCN’s standard-risk category into two separate entities: low-risk and intermediate-risk.

Low-risk group (non-*IKZF1*^plus^/MRD−), for the first time, was identified in adult *BCR::ABL1* + ALL. Prior studies indicated that *BCR::ABL1* + ALL patients attaining CMR at 3 months had favorable survival and did not benefit from transplantation [14, 15]. In contrast, the scenario for *IKZF1*^plus^/MRD− patients was distinct: despite achieving 3-month CMR, prognosis of these high-risk patients was still significantly worse than non-*IKZF1*^plus^ patients, and transplantation remarkably improved their OS (Supplemental Fig. 5G). This underscored that for *IKZF1*^plus^ genotype with an inherent propensity for relapse, achieving MRD negativity may not be sufficient to mitigate the adverse impact of a poor genetic profile. The classification of low-risk group was only restricted to non-*IKZF1*^plus^ patients who were MRD negative at 3 months, which did not benefit from allo-HSCT and should avoid it.

Within intermediate-risk group (non-*IKZF1*^plus^/MRD+), compared to non-HSCT subgroup, there was a promising trend towards better OS in allo-HSCT subgroup. It is advisable to employ targeted immunotherapies to eradicate MRD, and subsequently bridge allo-HSCT. To more comprehensively elucidate the role of allo-HSCT in this group, further studies with larger sample size and extended follow-up are warranted.

High-risk group actually consisted of all *IKZF1*^plus^ patients, irrespective of MRD at 3 months. Allo-HSCT subgroup consistently exhibited significantly better OS than non-HSCT counterparts, therefore allo-HSCT was crucial for high-risk patients by reducing relapse risk (Supplemental Fig. 5H).

In conclusion, the refined risk stratification based on *IKZF1*^plus^ genotyping and MRD assessment helps to guide transplantation decisions for adult *BCR::ABL1* + ALL patients. Allo-HSCT should be spared for the low-risk group, but is recommended for intermediate-risk and high-risk groups (Supplemental Fig. 6). More comprehensive clinical studies are warranted for further validation of our findings.

### Supplementary information

Supplemental Material

## Data Availability

Anonymized RNAseq data have been deposited in the Genome Sequence Archive for Humans (GSA-Human, https://ngdc.cncb.ac.cn/gsa-human) (accession number: HRA005804). For other data, please contact *jinwang@shsmu.edu.cn*.

## References

[1] Stanulla M, Dagdan E, Zaliova M, Möricke A, Palmi C, Cazzaniga G (2018). IKZF1plus defines a new minimal residual disease–dependent very-poor prognostic profile in pediatric b-cell precursor acute lymphoblastic leukemia. J Clin Oncol.

[2] National Comprehensive Cancer Network. NCCN clinical practice guidelines in oncology acute lymphoblastic leukemia. https://www.nccn.org/professionals/physician_gls/pdf/all.pdf.

[3] Ribera JM, Garcia O, Montesinos P, Brunet S, Abella E, Barrios M (2012). Treatment of young patients with Philadelphia chromosome-positive acute lymphoblastic leukaemia using increased dose of imatinib and deintensified chemotherapy before allogeneic stem cell transplantation. Br J Haematol.

[4] Chalandon Y, Thomas X, Hayette S, Cayuela JM, Abbal C, Huguet F (2015). Randomized study of reduced-intensity chemotherapy combined with imatinib in adults with Ph-positive acute lymphoblastic leukemia. Blood.

[5] Li JF, Dai YT, Lilljebjorn H, Shen SH, Cui BW, Bai L (2018). Transcriptional landscape of B cell precursor acute lymphoblastic leukemia based on an international study of 1,223 cases. Proc Natl Acad Sci USA.

[6] Liu W, Liu Y, Zhu Y, Weng X, Wang C, Ouyang W (2022). A phase ii study of flumatinib with chemotherapy for newly diagnosed Ph/BCR-ABL1-positive acute lymphoblastic leukemia in adults: preliminary results from RJ-ALL2020.2A trial. Blood.

[7] Liu W, Liu Y, Zhu Y, Weng X, Wang C, Ouyang W (2023). P363: a phase II study of flumatinib with chemotherapy for newly diagnosed Ph/BCR-ABL1-positive acute lymphoblastic leukemia in adults: updated results from RJ-ALL2020.2A trial. HemaSphere.

[8] Bastian L, Beder T, Barz MJ, Bendig S, Bartsch L, Walter W, et al. Developmental trajectories and cooperating genomic events define molecular subtypes of BCR::ABL1-positive ALL. Blood. 2023;143:1391–98. 10.1182/blood.2023021752 PMC11033585 38153913

[9] Kim JC, Chan-Seng-Yue M, Ge S, Zeng AGX, Ng K, Gan OI (2023). Transcriptomic classes of BCR-ABL1 lymphoblastic leukemia. Nat Genet.

[10] Beder T, Hansen BT, Hartmann AM, Zimmermann J, Amelunxen E, Wolgast N (2023). The gene expression classifier ALLCatchR identifies B-cell precursor ALL subtypes and underlying developmental trajectories across age. Hemasphere.

[11] Weng XQ, Shen Y, Sheng Y, Chen B, Wang JH, Li JM (2013). Prognostic significance of monitoring leukemia-associated immunophenotypes by eight-color flow cytometry in adult B-acute lymphoblastic leukemia. Blood Cancer J.

[12] Zhang Y, Jiang S, He F, Tian Y, Hu H, Gao L (2023). Single-cell transcriptomics reveals multiple chemoresistant properties in leukemic stem and progenitor cells in pediatric AML. Genome Biol.

[13] Aran D, Hu Z, Butte AJ (2017). xCell: digitally portraying the tissue cellular heterogeneity landscape. Genome Biol.

[14] Sasaki K, Kantarjian HM, Short NJ, Samra B, Khoury JD, Kanagal Shamanna R (2021). Prognostic factors for progression in patients with Philadelphia chromosome-positive acute lymphoblastic leukemia in complete molecular response within 3 months of therapy with tyrosine kinase inhibitors. Cancer.

[15] Ghobadi A, Slade M, Kantarjian H, Alvarenga J, Aldoss I, Mohammed KA (2022). The role of allogeneic transplant for adult Ph+ ALL in CR1 with complete molecular remission: a retrospective analysis. Blood.

