# Supplementary material for: Refined risk stratification helps guiding transplantation choice in adult BCR::ABL1-positive acute lymphoblastic leukemia
Source: Blood Cancer J. 2024 Apr 24;14(1):71. doi: 10.1038/s41408-024-01055-1 (PMC11043066; doi:10.1038/s41408-024-01055-1)
Supplement: Supplementary file 1 — Supplemental Material [file 41408_2024_1055_MOESM1_ESM.pdf]

Supplemental Appendix

Refined risk stratification helps guiding transplantation choice in adult *BCR::ABL1*-positive acute lymphoblastic leukemia

Supplementary Methods 2

Supplementary Results.. 5

Impact of *IKZF1* genotype or MRD status on survival outcomes 5

Survival outcomes of four subgroups combining both *IKZF1* genotype and MRD status 6

The role of allo-HSCT in *BCR::ABL1*+ ALL 7

Supplementary Figures.. 8

Supplemental Figure 1. Survival probability curves in TKI cohorts 8

Supplemental Figure 2. Genomic characteristics of *BCR::ABL1*+ ALL patients............................ 9

Supplemental Figure 3. Survival probability curves according to *IKZF1* genotype or MRD status at 3 months of treatment 11

Supplemental Figure 4. Survival probability curves among different groups 12

Supplemental Figure 5. Role of allo-HSCT in adult *BCR::ABL1*+ ALL 13

Supplemental Figure 6. Consort diagram of the refined risk stratification for adult *BCR::ABL1*+ ALL 14

Supplemental Figure 7. Cox proportional hazards regression analysis of survival 15

Supplemental Figure 8. Removal the batch effect of RNA sequencing data 16

Supplemental Figure 9. xCell analysis demonstrating reduced immune cell fractions in *IKZF1*^{plus} genotype.. 17

Supplementary Tables.. 19

Supplemental Table 1. Baseline characteristics and treatment response of 156 adult patients divided into imatinib and flumatinib group 19

Supplemental Table 2. Baseline characteristics and treatment response of 156 adult patients according to *IKZF1* genotype 21

Supplemental Table 3. Baseline characteristics and treatment response of 156 adult patients based on *IKZF1*^{plus} genotyping 23

Supplemental Table 4. Internal validation of MLPA technique 25

Supplemental Table 5. Baseline characteristics and treatment response of 128 patients stratified into three risk groups 27

Supplemental Table 6. Gene signatures of *IKZF1*^{plus} genotype in *BCR::ABL1*+ ALL 29

Supplementary Methods

Treatment protocols

Concurrent intensive chemotherapy and TKI proved to translate into high complete remission (CR) rates and improved survival. The Group for Research on Adult Acute Lymphoblastic Leukemia (GRAALL) randomly compared high doses of TKI combined with reduced-intensity chemotherapy to standard imatinib and hyper-CVAD (cyclophosphamide, vincristine, doxorubicin and dexamethasone) therapy. For patients receiving less-intense chemotherapy [*n* =135, median age: 48.6 years (range, 18-59)], the CR rate was 98.5% and estimated 5-year overall survival (OS) was 48.3% (95% CI, 39.2%-56.8%). Allo-HSCT in first CR prolonged relapse-free survival and overall survival. The induction regimen combining reduced-intensity chemotherapy (vincristine and dexamethasone) and imatinib (400mg/day) for *BCR::ABL1+ ALL* adult patients was validated in the GRAALPH-2005 study.(1)

Moreover, deintensified chemotherapy [including the induction regimen (VDP: vincristine, daunorubicin and prednisone) as well as one cycle of consolidation treatment (methotrexate, cytarabine, etoposide and mercaptopurine)] and increased dose of imatinib (600mg/day) proved feasible by the PETHEMA-ALL-Ph-08 Trial. All *BCR::ABL1*+ ALL adult patients [*n* = 29, mean age (standard deviation): 38 (9) years] achieved CR, and 90% underwent allo-HSCT. The 2-year event-free survival (EFS) was 63% (95% CI, 39%-87%).(2)

Therefore, in order to reduce high-dose chemotherapy-associated toxicity and early death rate, for 156 patients with written informed consent who were treated in our constitution, we employed imatinib (600mg/day) or flumatinib (600mg/day) combined with relative low-intensity VDP-based induction chemotherapy (vincristine, daunorubicin and prednisone), and consolidation therapy [VAD (vincristine, adriamycin and dexamethasone), 4 cycles for patients transplanted and 8 cycles

for not transplanted]. Additionally, patients without transplantation followed 2 years maintenance therapy [POMP (prednisone, vincristine, methotrexate, and mercaptopurine)]. While the triple intrathecal therapy was intensified in order to prevent central nervous system leukemia, at least 6 times before allo-HSCT and up to 16 times for those without allo-HSCT.

As to blinatumomab, at the time of registration of our three clinical trials (July 8, 2014, January 17, 2021 and March 14, 2021), blinatumomab had not been available in China (approval date: August 19, 2021). Thus, *BCR::ABL1*+ ALL patients in this study were treated with a combination of first or second-generation TKI, multiagent chemotherapy and allo-HSCT, without incorporating blinatumomab as frontline therapy.

Copy number variation analysis

Fresh bone marrow (*n* = 154) and peripheral blood (*n* = 2) samples were collected from patients at diagnosis. Copy number variations (CNVs) were detected by multiplex ligation-dependent probe amplification (MLPA) using SALSA MLPA P335-C2, P202-C1 and P419-B1 kits (MRC Holland, Amsterdam, The Netherlands). Genetic analysis was performed as previously described.(3-5) MLPA also allowed the assessment of *IKZF1* isoforms, including wildtype, dominant-negative, haploinsufficiency, and miscellaneous subtypes.(6)

To further validate MLPA results, new whole genome sequencing (WGS) analyses were additionally performed on 23 diagnostic (mean sequencing depth of 40×) and 23 matched remission (mean sequencing depth of 10×) DNA samples. The 23 patients include 7 no *IKZF1* deletion patients, 8 *IKZF1* deletion only patients and 8 $IKZF1^{plus}$ patients. The tumor sample's WGS FASTQ files were aligned to the human hg19 reference genome using BWA MEM (v0.7.17-r1188). The resulting BAM files were processed with GATK (v4.3.0.0) using the MarkDuplicates

subcommand to mark PCR duplicates. The marked BAM files were subjected to CNV detection using the Tumor-only mode of HMFtools' purple (v4.0.2) software. To enhance sensitivity of the CNV detection results, R packages ExomeDepth (v1.1.6) was also employed for CNV detection. We also used Manta (v1.6.0) to call for deletion events.

Response Assessment and Definitions

CR was defined as the presence of less than 5% blasts in bone marrow, with an absolute neutrophil count ≥ 1 × 10^9/L and platelet count ≥ 100 × 10^9/L in peripheral blood and no extramedullary disease. CR with incomplete hematologic recovery (CRi) was defined as a CR but with an absolute neutrophil count < 1 × 10^9/L, or platelets < 100 × 10^9/L in peripheral blood. Relapse was defined as the recurrence of over 5% lymphoblasts in bone marrow or by the presence of extramedullary disease after CR. Multiparameter flow cytometry (MFC) was performed for antigen expression as described previously(7). Real-time quantitative polymerase chain reaction (RT-qPCR) was employed to routinely monitor MRD. MRD negativity, equivalent to CMR in this study, was defined as the absence of detectable *BCR::ABL1* transcript by RT-qPCR (< 0.001%). MRD positivity was defined as the failure to achieve CMR.

Next-generation sequencing

Targeted exome sequencing (*n* = 105, including 35 *IKZF1*^{plus} patients) and RNA sequencing (*n* = 137, including 49 *IKZF1*^{plus} patients) were performed with patients' samples at diagnosis, as we previously reported.(8, 9)

Statistical Analysis

OS was calculated from diagnosis to death or last follow-up. EFS was defined as time from diagnosis to an event (including induction failure, relapse, or death) or last follow-up. Relapse-free survival (RFS) was considered as time from CR to relapse or death. Cumulative incidence of relapse (CIR) was defined as time from CR to the first relapse, considering death in CR as a competing event. OS and EFS were both estimated using the Kaplan-Meier method and log-rank test, while CIR was compared with Gray's method. The Chi-square test or Fisher exact test was used to examine statistical significance of categorical variables. The Cox proportional hazards regression model was applied for prognostic factors. All reported P values were 2-sided, and P values < 0.05 were considered statistically significant. All statistical analyses were conducted using R statistical software, version 4.2.3 (https://www.r-project. org/).

Supplementary Results

Impact of *IKZF1* genotype or MRD status on survival outcomes

IKZF1^{plus} group ($n = 48$) had 3-year OS, EFS, and CIR rates of 52.1% (38.2%–70.9%), 34.7% (22.4%–53.7%), and 56.6% (39.1%–70.8%), respectively. While non-*IKZF1*^{plus} group ($n = 91$) had 3-year OS, EFS, and CIR rates of 77.5% (68.1%–88.1%), 61.3% (51.2%–73.5%), and 29.2% (19.2%–40.0%), respectively **(Supplemental Fig. 3A, B, C)**.

Simultaneously, MRD status is a well-recognized risk factor in ALL. Patients who failed to achieve CMR at 3 months (MRD+, $n = 68$) had 3-year OS, EFS, and CIR rates of 56.6% (44.6%–71.9%), 41.0% (29.9%–56.2%), and 46.8% (33.2%–59.4%), respectively. While patients who attained CMR at 3 months (MRD-, $n = 57$), with 3-year OS, EFS, and CIR rates of 80.7% (70.0%–93.1%), 61.1% (48.2%–77.4%), and 31.2% (17.9%-45.4%), respectively **(Supplemental Fig. 3D, E, F)**.

Survival outcomes of four subgroups combining both *IKZF1* genotype and MRD status

The 3-year OS rate of non-*IKZF1*^{plus}/MRD- subgroup [87.0% (75.1%-99.0%)] was the best, higher than the other three subgroups **(Supplemental Fig. 4A)**. Pairwise comparison showed that there was no significant difference between *IKZF1*^{plus}/MRD- and *IKZF1*^{plus}/MRD+ subgroups (P = 0.12), suggesting that *IKZF1*^{plus} patients had very poor outcomes regardless of MRD status in long term.

Furthermore, 3-year EFS rate of non-*IKZF1*^{plus}/MRD- [75.2% (59.7%-90.7%)] subgroup was also the highest, higher than non-*IKZF1*^{plus}/MRD+ [46.5% (29.4%-63.6%)], *IKZF1*^{plus}/MRD- [32.3% (6.6%-58.0%)], or *IKZF1*^{plus}/MRD+ [32.0% (13.7%-50.3%)] subgroup, respectively **(Supplemental Fig. 4B)**. Pairwise comparison revealed that there was also no significant difference between *IKZF1*^{plus}/MRD- and *IKZF1*^{plus}/MRD+ subgroups (P = 0.14), further demonstrating the dismal prognosis of *IKZF1*^{plus} patients.

Notably, 3-year CIR rate of non-*IKZF1*^{plus}/MRD- subgroup [15.5% (5.2%-30.9%)] was the lowest, compared to that of non-*IKZF1*^{plus}/MRD+ [41.9% (24.4%-58.4%)], *IKZF1*^{plus}/MRD- [62.7% (28.5%-84.1%)] or *IKZF1*^{plus}/MRD+ [56.0% (34.0%-73.2%)] subgroup, respectively **(Supplemental Fig. 4C)**. Pairwise comparison showed that there still was no significant difference between *IKZF1*^{plus}/MRD- and *IKZF1*^{plus}/MRD+ subgroups (P = 0.31). This confirmed that more than half of *IKZF1*^{plus} patients tended to relapse despite MRD negativity, resulting in impaired OS and EFS.

Two *IKZF1*^{plus} subgroups had the worst outcomes and highest relapse rates, therefore combined into high-risk group (*IKZF1*^{plus}, MRD+ or MRD-) (n = 45) **(Supplemental Fig. 4D-F)**.

The role of allo-HSCT in *BCR::ABL1*+ ALL

Initially, solely according to MRD status at 3 months, 125 patients (aged 18-64 years) eligible for transplantation were analyzed. Among patients who were MRD-, there was a reversal in 2-year and 3-year OS rates between allo-HSCT and non-HSCT groups **(Supplemental Fig. 5A)**. This suggested that MRD status alone was not sufficient to predict prognosis, and other factors needed to be combined for better risk stratification. Meanwhile, among patients who were MRD+, 3-year OS of allo-HSCT group [81.2% (69.5%-95.0%)] was significantly higher than non-HSCT group [22.1% (9.9%-49.3%)] **(Supplemental Fig. 5B)**. This showed that adult patients who were still MRD positive at 3 months benefited significantly from allo-HSCT.

Next, solely according to *IKZF1* genotype, 139 patients (aged 18-64 years) eligible for transplantation were analyzed. Among non-*IKZF1*^{plus} patients, two groups exhibited inverse 2-year and 3-year OS rates **(Supplemental. 5C)**. This suggested that *IKZF1* genotype alone was not enough to predict the prognosis, and refinement of risk stratification was needed. Simultaneously, among *IKZF1*^{plus} patients, 3-year OS of allo-HSCT group [80.3% (64.0%-100.0%)] was significantly higher than non-HSCT group [23.3% (10.2%-53.2%)] **(Supplemental. 5D)**. This confirmed that adult patients with *IKZF1*^{plus} genotype notably benefited from allo-HSCT.

Supplementary Figures

Supplemental Figure 1. Survival probability curves in TKI cohorts

(ABC) Overall survival, event-free survival and cumulative incidence of relapse for patients grouped by TKI. *MRD- refers to patients achieving CMR (evaluated by RT-qPCR with a sensitivity of 0.001%) at 3 months of treatment, while MRD+ refers to patients failing to achieve CMR at 3 months.

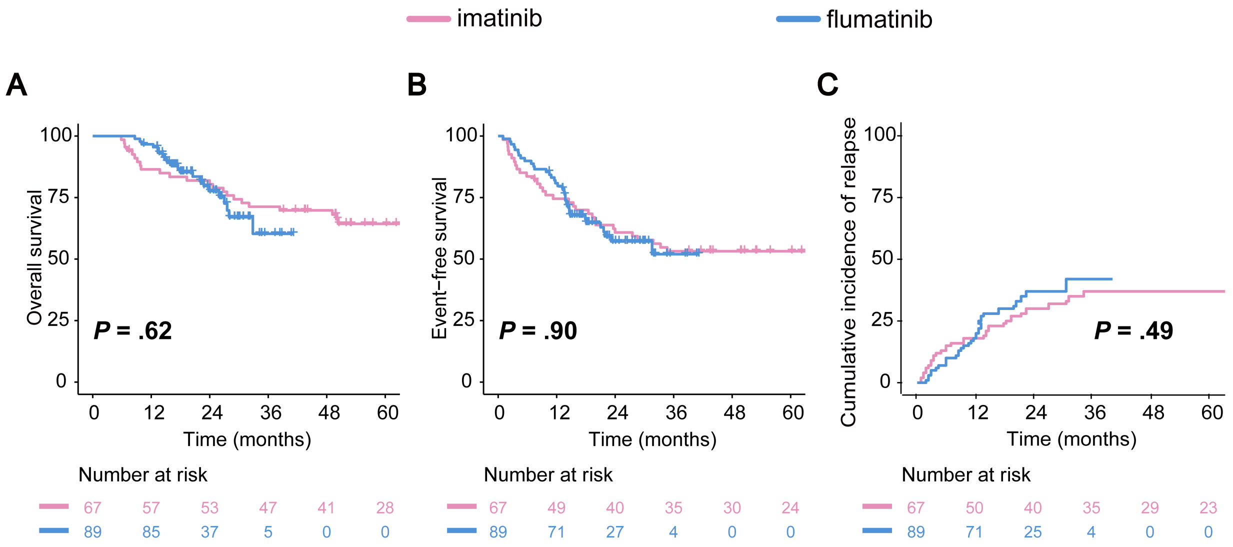

Supplemental Figure 2. Genomic characteristics of *BCR::ABL1*+ ALL patients

(A) The distribution of *IKZF1* exon deletions in *IKZF1*^{plus} and *IKZF1* deletion alone groups. (B) Correlation between clinical characteristics and genetic alterations (*n* = 153, 3 patients without flow cytometry results). Asterisks refer to statistically significant correlations. *P* values are calculated using Fisher exact test. *$P < 0.05$, ** $P < 0.01$, *** $P < 0.005$, and **** $P < 0.001$. (C) Use whole genome sequencing (WGS) to validate *IKZF1, CDKN2A, CDKN2B, PAX5,* and *ERG* deletions detected by multiplex ligation-dependent probe amplification (MLPA). Among 23 *BCR::ABL1*+ ALL patients (7 no *IKZF1* deletion patients, 8 *IKZF1* deletion only patients and 8 *IKZF1*^{plus} patients), the concordant rate for copy number variations (CNVs) was 82.6% (19/23). Meanwhile, the concordant rate for deletions in the 5 genes was 95.7% [110/(23x5)], and the 5 inconsistent pairs of deletions (4.3%) were highlighted in red rectangles.

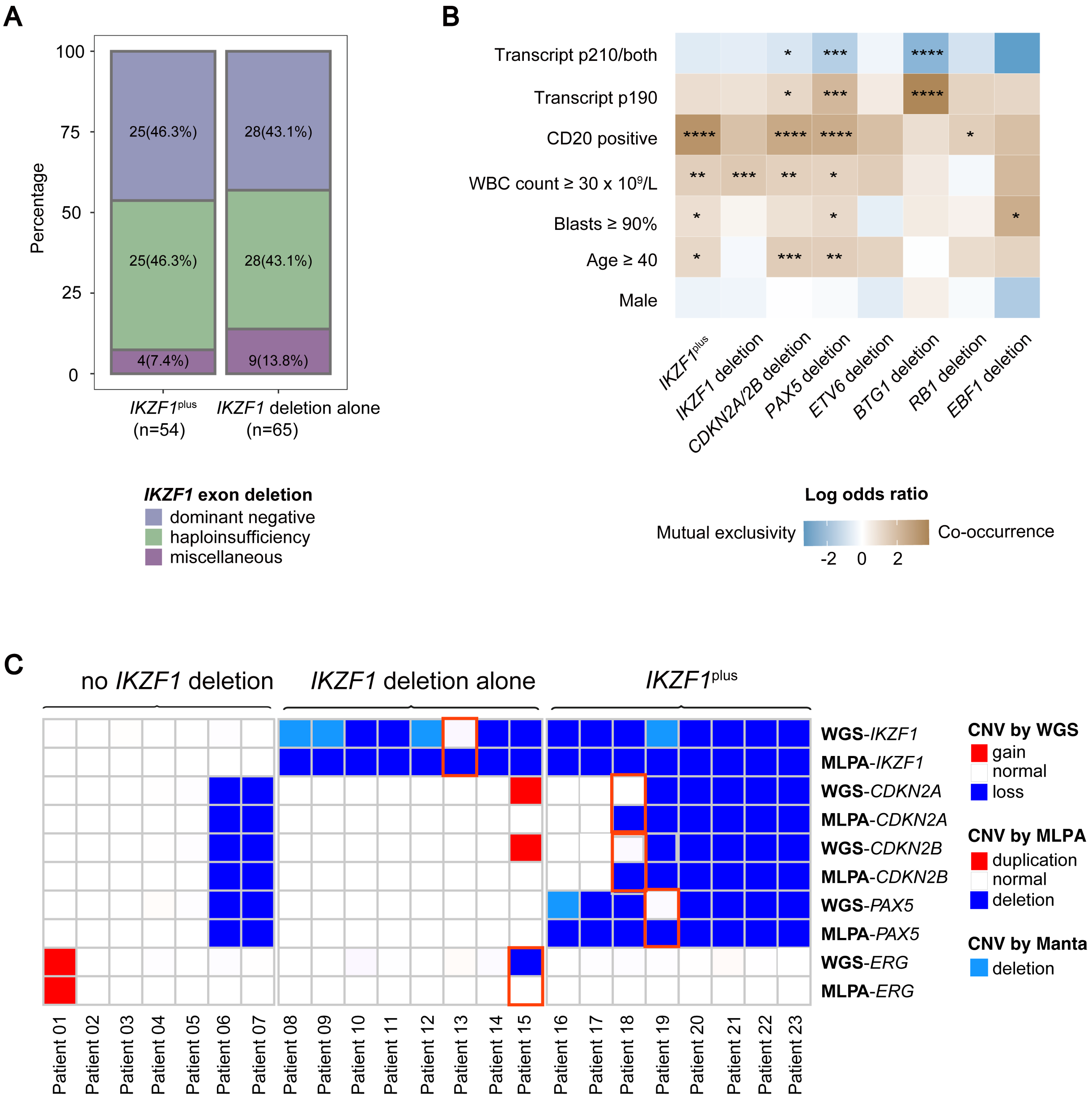
A
Percentage
100
75
50
25
0
25(46.3%)
28(43.1%)
25(46.3%)
28(43.1%)
4(7.4%)
9(13.8%)
IKZF1plus (n=54)
IKZF1 deletion alone (n=65)
IKZF1 exon deletion
dominant negative
haploinsufficiency
miscellaneous
B
Transcript p210/both
Transcript p190
CD20 positive
WBC count ≥ 30 x 10⁹/L
Blasts ≥ 90%
Age ≥ 40
Male
IKZF1plus
IKZF1 deletion
CDKN2A/2B deletion
PAX5 deletion
ETV6 deletion
BTG1 deletion
RB1 deletion
EBF1 deletion
Log odds ratio
Mutual exclusivity
Co-occurrence
-2 0 2
C
no IKZF1 deletion
IKZF1 deletion alone
IKZF1plus
WGS-IKZF1
MLPA-IKZF1
WGS-CDKN2A
MLPA-CDKN2A
WGS-CDKN2B
MLPA-CDKN2B
WGS-PAX5
MLPA-PAX5
WGS-ERG
MLPA-ERG
CNV by WGS
gain
normal
loss
CNV by MLPA
duplication
normal
deletion
CNV by Manta
deletion
Patient 01
Patient 02
Patient 03
Patient 04
Patient 05
Patient 06
Patient 07
Patient 08
Patient 09
Patient 10
Patient 11
Patient 12
Patient 13
Patient 14
Patient 15
Patient 16
Patient 17
Patient 18
Patient 19
Patient 20
Patient 21
Patient 22
Patient 23

Supplemental Figure 3. Survival probability curves according to *IKZF1* genotype or MRD status at 3 months of treatment

Overall survival, event-free survival and cumulative incidence of relapse for patients (18-64 years) grouped by (ABC) MRD status at 3 months (MRD+ or MRD-), and (DEF) *IKZF1* genotype (*IKZF1*^{plus} or non-*IKZF1*^{plus}), respectively. *MRD- refers to patients achieving CMR (evaluated by RT-qPCR with a sensitivity of 0.001%) at 3 months of treatment, while MRD+ refers to patients failing to achieve CMR at 3 months.

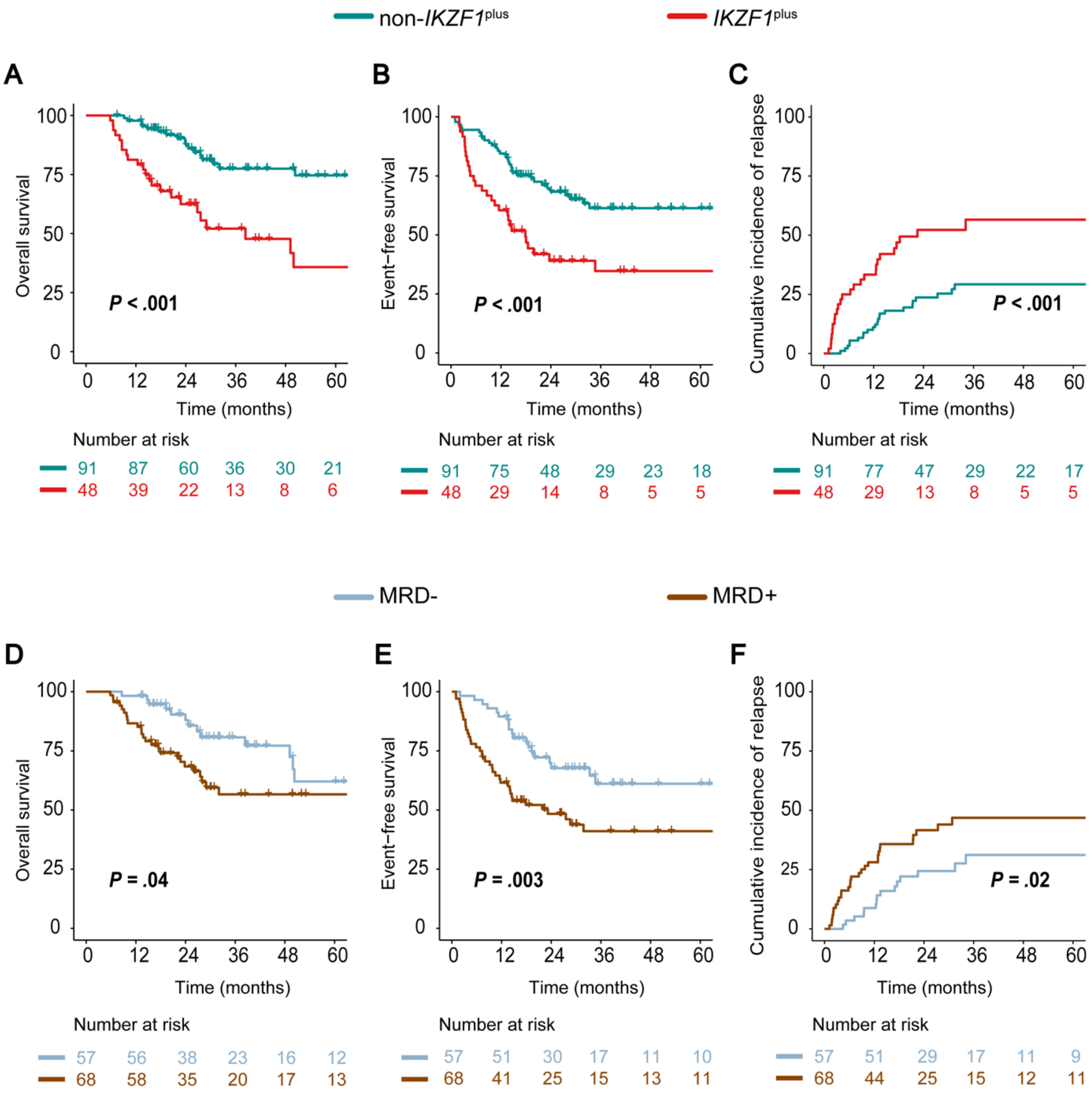

Supplemental Figure 4. Survival probability curves among different groups

Overall survival, event-free survival and cumulative incidence of relapse for patients (18-64 years) (ABC) grouped by both *IKZF1* genotype and MRD status at 3 months or (DEF) stratified into three risk groups. *IKZF1*plus/MRD- and *IKZF1*plus/MRD+ subgroups, who have the worst outcomes and highest relapse rates, are combined as high-risk group (*IKZF1*plus, MRD+ or MRD-) due to no significant differences observed. *MRD- refers to patients achieving CMR (evaluated by RT-qPCR with a sensitivity of 0.001%) at 3 months of treatment, while MRD+ refers to patients failing to achieve CMR at 3 months.

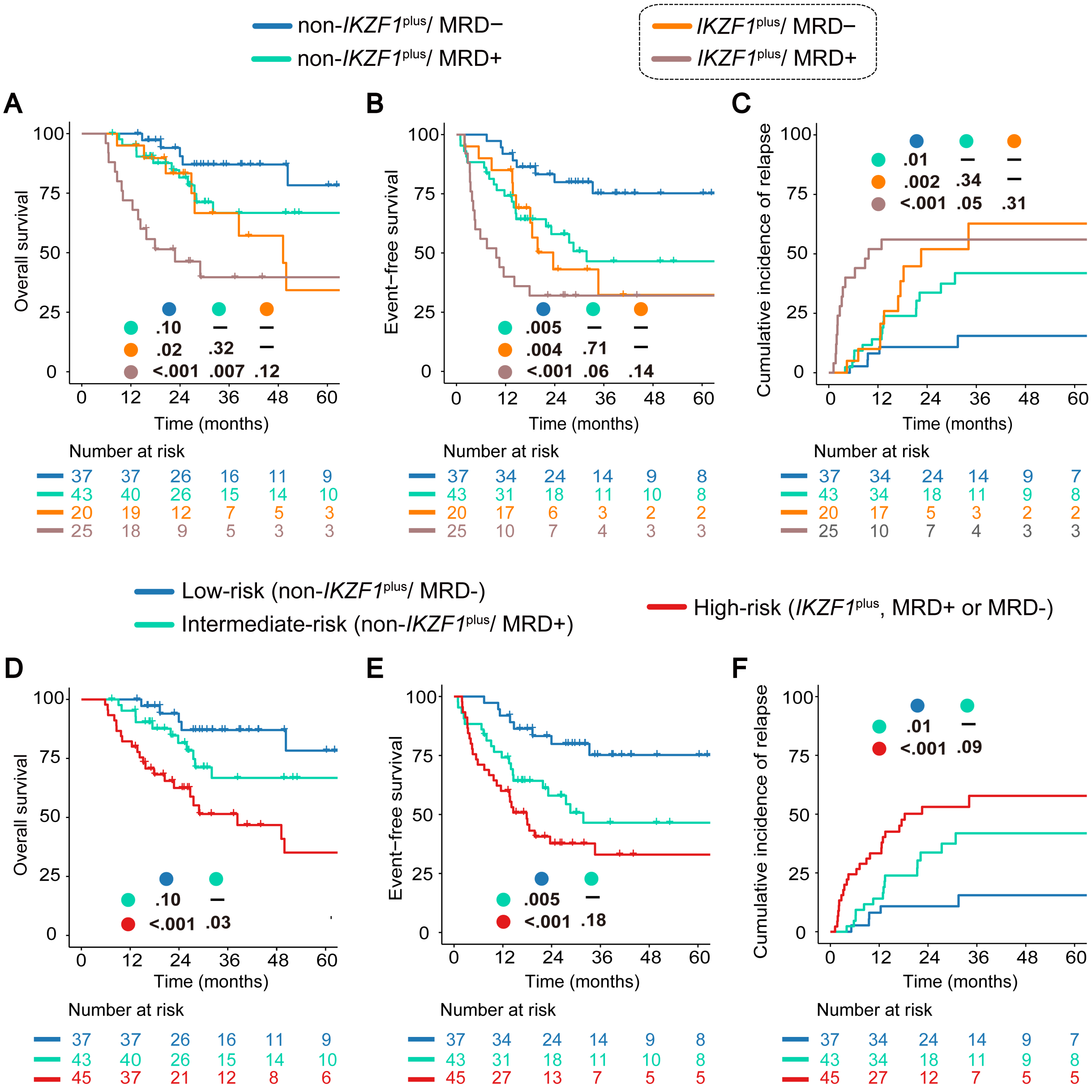

Supplemental Figure 5. Role of allo-HSCT in adult *BCR::ABL1*+ ALL

Effects of transplantation on overall survival of patients (18-64 years) who are (A) MRD- at 3 months, (B) MRD+ at 3 months, (C) non-*IKZF1*^{plus}, (D) *IKZF1*^{plus}, respectively. (EF) Overall survival of three risk groups without or undergoing allo-HSCT. (G) Overall survival of patients undergoing or without allo-HSCT in *IKZF1*^{plus}/MRD- subgroup. (H) Cumulative incidence of relapse of patients undergoing or without allo-HSCT in high-risk group. *P* values are calculated using the log-rank test. The numbers at risk are based on all evaluable patients. *MRD- refers to patients achieving CMR (evaluated by RT-qPCR with a sensitivity of 0.001%) at 3 months of treatment, while MRD+ refers to patients failing to achieve CMR at 3 months.

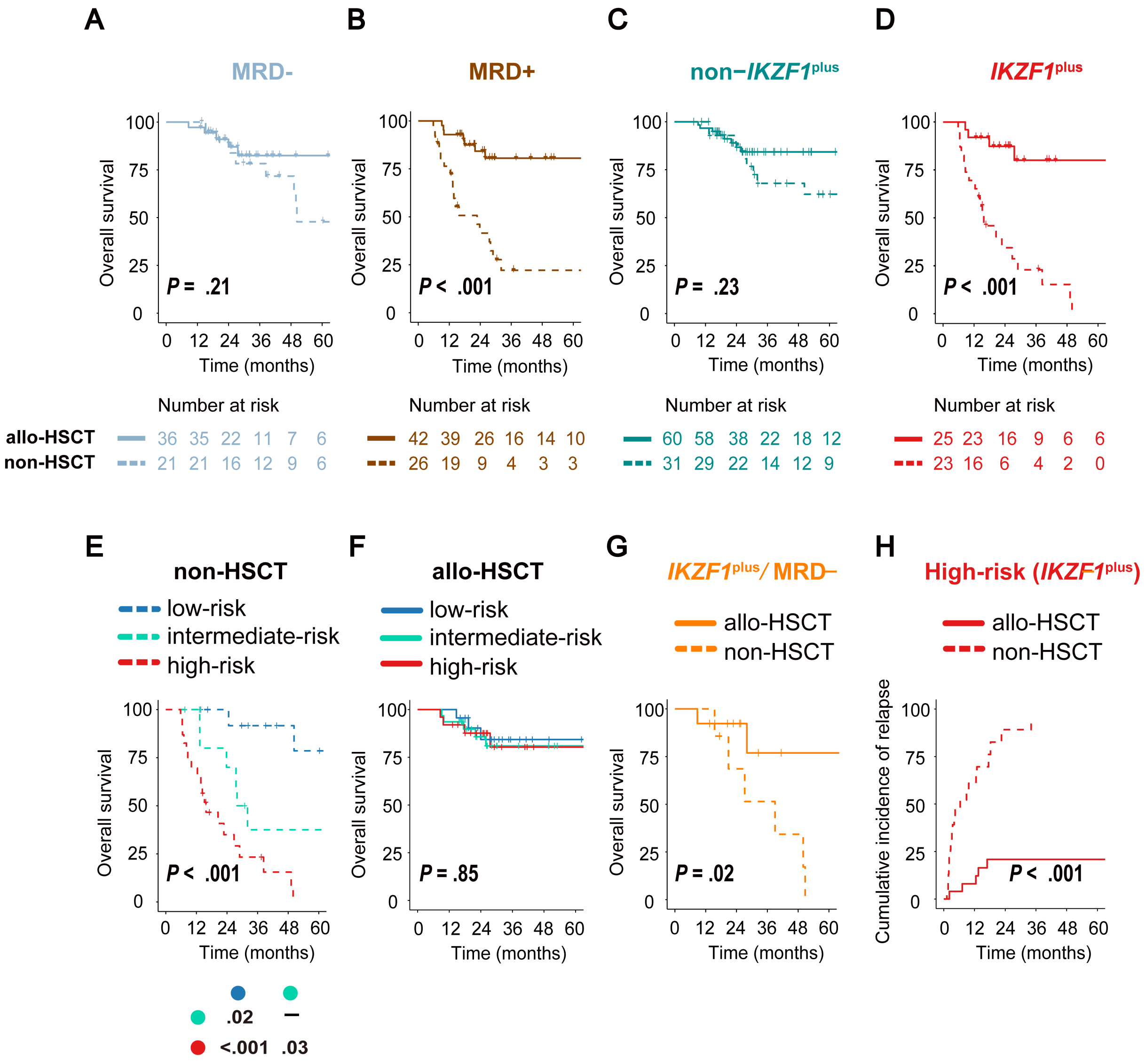

Supplemental Figure 6. Consort diagram of the refined risk stratification for adult *BCR::ABL1*+ ALL

MRD- refers to patients achieving CMR (evaluated by RT-qPCR with a sensitivity of 0.001%) at 3 months of treatment, while MRD+ refers to patients failing to achieve CMR at 3 months. The patients without MRD evaluation at 3 months of treatment are classified as not available. *IKZF1*^{plus}/MRD-, *IKZF1*^{plus}/MRD+ and *IKZF1*^{plus}/MRD not available (n = 3) subgroups have the worst outcomes and highest relapse rates, therefore combined into the high-risk group (*IKZF1*^{plus}, n = 48), indicating MRD assessment not contributing to risk stratification of *IKZF1*^{plus} patients.

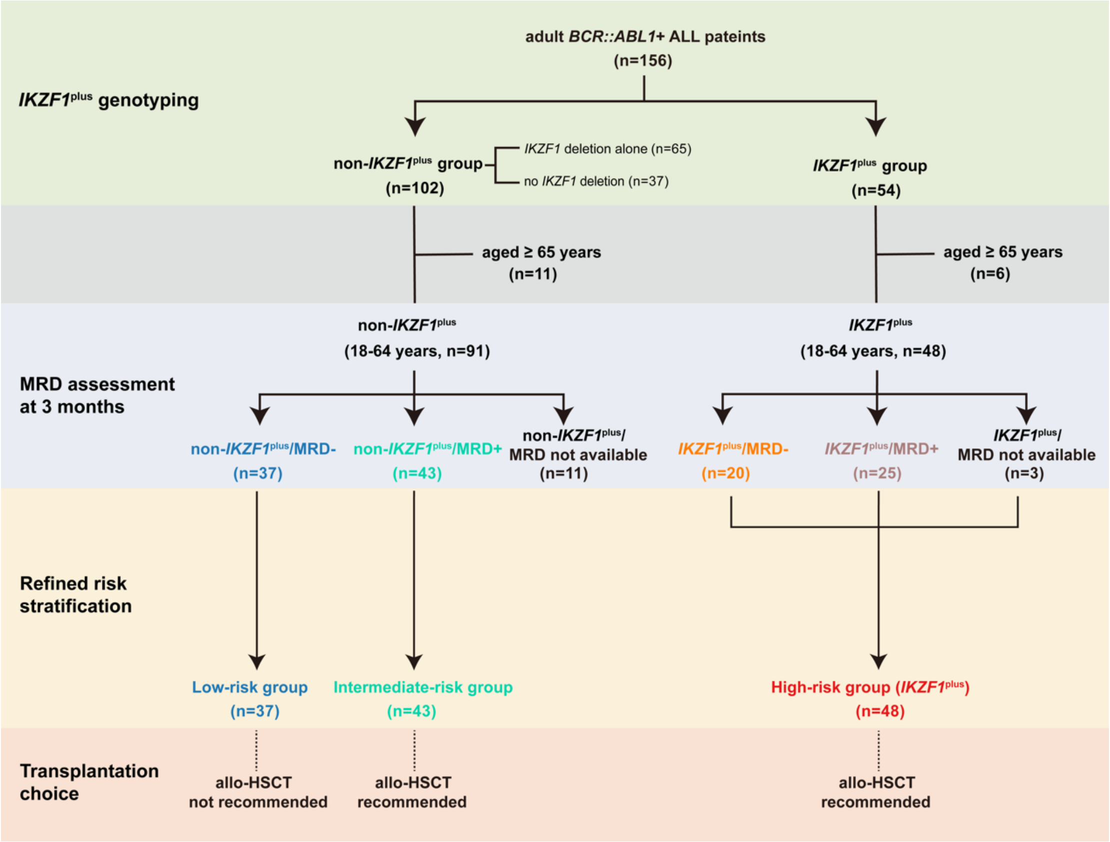

Supplemental Figure 7. Cox proportional hazards regression analysis of survival

Forest plot exhibits univariate and multivariate analysis of overall survival, event-free survival and relapse-free survival. HRs and corresponding 95% CIs are listed next to each variable, depicted as diamonds and horizontal lines. The vertical lines intersecting the value of 1 represent non-statistically significant effect, while values exceeding 1 indicate adverse effects. *MRD- refers to patients achieving CMR (evaluated by RT-qPCR with a sensitivity of 0.001%) at 3 months of treatment, while MRD+ refers to patients failing to achieve CMR at 3 months. Abbreviations: ACA, additional chromosomal abnormalities; Ph, Philadelphia chromosome; CR, complete remission; CRi, CR with incomplete hematologic recovery; EOI, end of induction; HR, hazard ratio; CI, confidence interval.

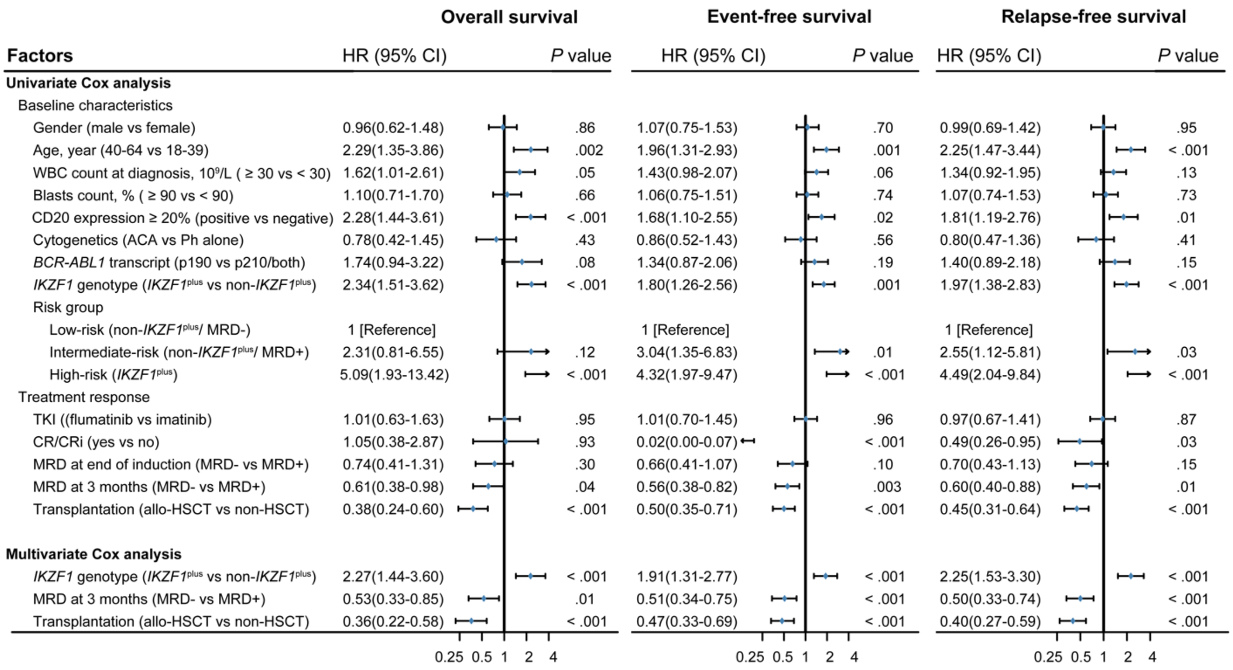

Supplemental Figure 8. Removal the batch effect of RNA sequencing data

Three kinds of colors refer to three different sequencing platforms, and points represent samples. PCA is applied to various gene sets, including all genes, top 5% of all genes with the largest variance, protein-coding genes, and top 5% high variance protein-coding genes, both before and after SVA adjustment. PCA, principal component analysis; SVA, surrogate variable analysis.

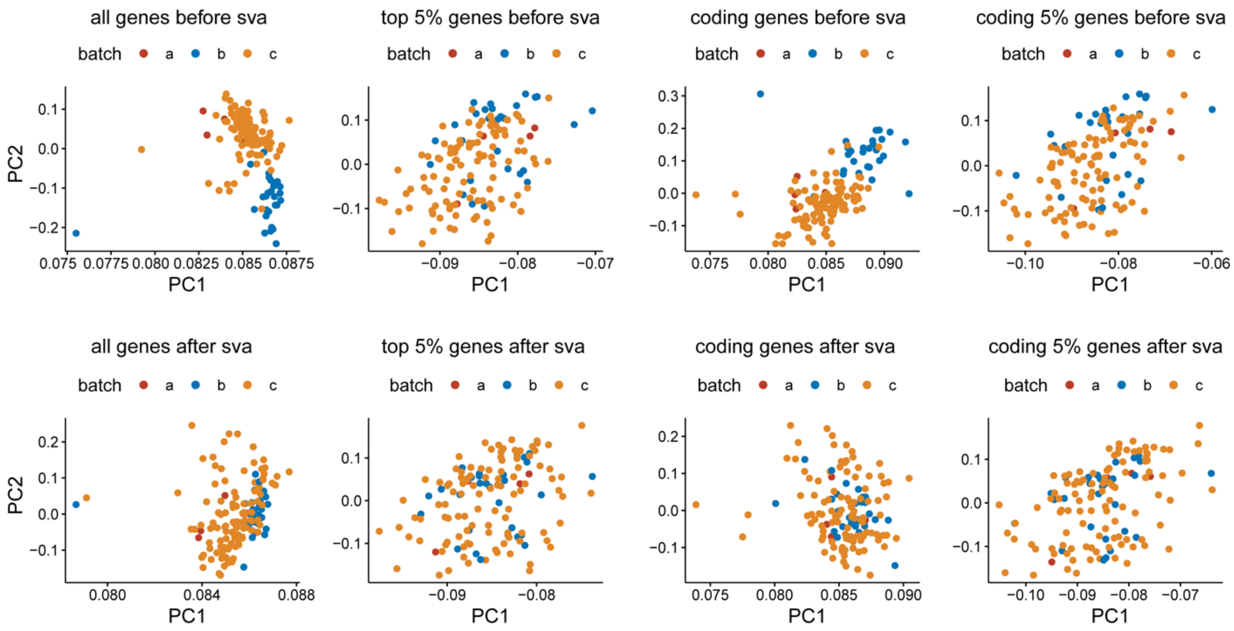

Supplemental Figure 9. xCell analysis demonstrating reduced immune cell fractions in *IKZF1*^{plus} genotype

(A) Bulk RNA sequencing-based immunocyte deconvolution analysis shows proportions of diverse cell types between *IKZF1*^{plus} and non-*IKZF1*^{plus} group using xCell method. Abbreviations: HSC, hematopoietic stem cell; CMP, common myeloid progenitor; GMP, granulocyte–macrophage progenitor; MEP, megakaryocyte and erythroid progenitor; Tcm, central memory T cell; Tem, effector memory T cell. (B) Box plots illustrating expression of CD3 antigen between *IKZF1*^{plus} and non-*IKZF1*^{plus} group, as measured by flow cytometry (n = 123, 14 patients without results). P values are calculated using Wilcoxon rank-sum test. *$P < 0.05$, ** $P < 0.01$, *** $P < 0.005$, and **** $P < 0.001$.

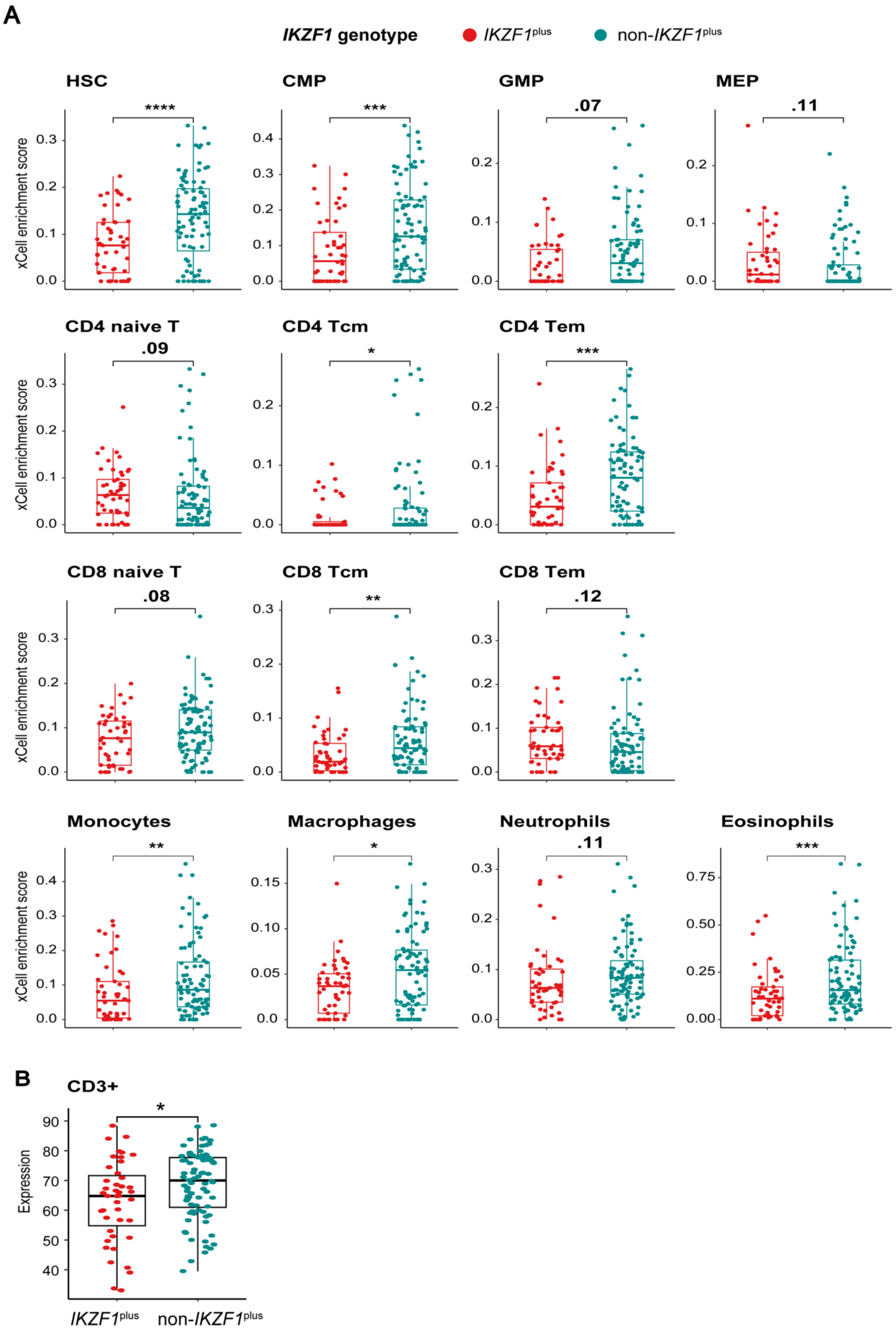
A
IKZF1 genotype
IKZF1plus
non-IKZF1plus
HSC
****
CMP
***
GMP
.07
MEP
.11
CD4 naive T
.09
CD4 Tcm
*
CD4 Tem
***
CD8 naive T
.08
CD8 Tcm
**
CD8 Tem
.12
Monocytes
**
Macrophages
*
Neutrophils
.11
Eosinophils
***
xCell enrichment score
B
CD3+
*
Expression
IKZF1plus
non-IKZF1plus

Supplementary Tables

Supplemental Table 1. Baseline characteristics and treatment response of 156 adult patients divided into imatinib and flumatinib group

	imatinib(N=67)*	flumatinib(N=89)*	*P* value†
**Median follow-up period (range), months**	72.8 (7.5-116.5)	24.8 (10.5-41.2)	
**Baseline characteristics**			
Gender			
Male	36 (53.7%)	48 (53.9%)	1.00
Female	31 (46.3%)	41 (46.1%)	
Age at diagnosis, years			
Median (range)	46 (19-71)	50(19-76)	
18-39	28 (41.8%)	30 (33.7%)	0.07
40-64	36 (53.7%)	45 (50.6%)	
≥ 65	3 (4.5%)	14 (15.7%)	
WBC count at diagnosis, x10^9/L			
Median (IQR)	36 (14.8-74.4)	43 (16.1-101.7)	
< 30	30 (44.8%)	31 (34.8%)	0.25
≥ 30	37 (55.2%)	58 (65.2%)	
Blasts count at diagnosis, %			
Median (IQR)	88.9 (74.0-93.5)	90.7 (81.0-94.5)	
< 90	36 (53.7%)	39 (43.8%)	0.26
≥ 90	31 (46.3%)	50 (56.2%)	
CD19 expression ≥ 20%			
Patients with available data	66 (98.5%)	87 (97.8%)	
Positive	66 (100%)	87 (100%)	1.00
Negative	0 (0%)	0 (0%)	
CD20 expression ≥ 20%			
Patients with available data	66 (98.5%)	87 (97.8%)	
Positive	12 (18.2%)	12 (13.8%)	0.51
Negative	54 (81.8%)	75 (86.2%)	
Cytogenetics at diagnosis			
Normal	15 (22.4%)	15 (16.9%)	0.53
Isolated Ph	17 (25.4%)	17 (19.1%)	
ACA	26 (38.8%)	43 (48.3%)	
Unknown	9 (13.4%)	14 (15.7%)	
CNS involvement			
Yes	4 (6.0%)	4 (4.5%)	0.73
No	63 (94.0%)	85 (95.5%)	
BCR::ABL1 transcript			
p190	50 (74.6%)	65 (73.0%)	1.00
p210	12 (17.9%)	17 (19.1%)	
p190 and p210	5 (7.5%)	7 (7.9%)	

**Treatment response**			
CR/CRi at EOI			
Yes	63 (94.0%)	86 (96.6%)	0.46
No	4 (6.0%)	3 (3.4%)	
MRD status at EOI, RT-qPCR			
Patients with available data	66 (98.5%)	85 (95.5%)	
MRD-	13 (19.7%)	21 (24.7%)	0.56
MRD+	53 (80.3%)	64 (75.3%)	
MRD status at 3 months, RT-qPCR			
Patients with available data	58 (86.6%)	83 (93.3%)	
MRD-	26 (44.8%)	36 (43.4%)	0.86
MRD+	32 (55.2%)	47 (56.6%)	
BCR::ABL1 mutation status			
Patients with available data	51 (76.1%)	89 (100%)	
Mutation	19 (37.3%)	41 (46.1%)	0.38
No mutation detected	32 (62.7%)	48 (53.9%)	
Transplantation			
Patients with available data	64 (95.5%)	75 (84.3%)	
Allo-HSCT	33 (51.6%)	52 (69.3%)	0.04
Non-HSCT	31 (48.4%)	23 (30.7%)	
Relapse	26 (38.8%)	30 (33.7%)	0.61
Death	24 (35.8%)	21 (23.6%)	0.11

Note: WBC: white blood cell; IQR: interquartile range; ACA: additional chromosomal abnormalities; Ph: Philadelphia chromosome; CNS: central nervous system; CR: complete remission; CRi: CR with incomplete hematologic recovery; EOI: end of induction; MRD: minimal residual disease; RT-qPCR: real-time quantitative polymerase chain reaction; Allo-HSCT: allogenic hematopoietic stem cell transplantation.

* Data are n (%).

† Fisher exact tests were performed to compare categorical variables between cohorts.

Supplemental Table 2. Baseline characteristics and treatment response of 156 adult patients according to *IKZF1* genotype

	IKZF1*plus (N=54)	***IKZF1* deletion alone (N=65)***	**no *IKZF1* deletion (N=37)***	***P* value†**
**Baseline characteristics**				
Gender				
Male	26 (48.1%)	37 (56.9%)	21 (56.8%)	0.58
Female	28 (51.9%)	28 (43.1%)	16 (43.2%)	
Age at diagnosis, years				
Median (range)	50 (20-75)	40 (19-76)	49 (20-71)	
18-39	14 (25.9%)	32 (49.2%)	12 (32.4%)	0.03
40-64	34 (63.0%)	24 (36.9%)	23 (62.2%)	
≥ 65	6 (11.1%)	9 (13.8%)	2 (5.4%)	
WBC count at diagnosis, x10^9/L				
Median (IQR)	51.5 (30.9-130.2)	38.7 (17.0-85.6)	21.2 (6.3-67.9)	
< 30	13 (24.1%)	26 (40.0%)	22 (59.5%)	0.003
≥ 30	41 (75.9%)	39 (60.0%)	15 (40.5%)	
Blasts count at diagnosis, %				
Median (IQR)	91.3(85.0-94.4)	88.5 (81.0-94.0)	84.0(70.0-93.0)	
< 90	20 (37.0%)	35 (53.8%)	20 (54.1%)	0.13
≥ 90	34 (63.0%)	30 (46.2%)	17 (45.9%)	
CD19 expression ≥ 20%				
Patients with available data	54 (100%)	65 (100%)	34 (91.2%)	
Positive	54 (100%)	65 (100%)	34 (100%)	1.00
Negative	0 (0%)	0 (0%)	0 (0%)	
CD20 expression ≥ 20%				
Patients with available data	54 (100%)	65 (100%)	34 (91.2%)	
Positive	19 (35.2%)	3 (4.6%)	2 (5.9%)	<0.001
Negative	35 (64.8%)	62 (95.4%)	32 (94.1%)	
Cytogenetics at diagnosis				
Normal	9 (16.7%)	9 (13.8%)	12 (32.4%)	0.42
Isolated Ph	12 (22.2%)	16 (24.6%)	6 (16.2%)	
ACA	24 (44.4%)	30 (46.2%)	15 (40.5%)	
Unknown	9 (16.7%)	10 (15.4%)	4 (10.8%)	
CNS involvement				
Yes	2 (3.7%)	6 (9.2%)	0 (0%)	0.11
No	52 (96.3%)	59 (90.8%)	37 (100%)	
BCR::ABL1 transcript				
p190	44 (81.5%)	47 (72.3%)	24 (64.9%)	0.42
p210	8 (14.8%)	12 (18.5%)	9 (24.3%)	
p190 and p210	2 (3.7%)	6 (9.2%)	4 (10.8%)	

**Treatment response**				
Tyrosine kinase inhibitor				
Imatinib	23 (42.6%)	27 (41.5%)	17 (45.9%)	0.91
Flumatinib	31 (57.4%)	38 (58.5%)	20 (54.1%)	
CR/CRi at EOI				
Yes	52 (96.3%)	60 (92.3%)	37 (100%)	0.19
No	2 (3.7%)	5 (7.7%)	0 (0%)	
MRD status at EOI, RT-qPCR				
Patients with available data	53 (98.1%)	64 (98.5%)	34 (91.9%)	
MRD-	14 (26.4%)	14 (21.9%)	6 (17.6%)	0.62
MRD+	39 (73.6%)	50 (78.1%)	28 (82.4%)	
MRD status at 3 months, RT-qPCR				
Patients with available data	51 (94.4%)	57 (87.7%)	33 (89.2%)	
MRD-	24 (47.1%)	20 (35.1%)	18 (54.5%)	0.17
MRD+	27 (52.9%)	37 (64.9%)	15 (45.5%)	
BCR::ABL1 mutation status				
Patients with available data	49 (90.7%)	57 (87.7%)	34 (91.9%)	
Mutation	23 (46.9%)	25 (43.9%)	12 (35.3%)	0.56
No mutation detected	26 (53.1%)	32 (56.1%)	22 (64.7%)	
Transplantation				
Patients with available data	48 (88.9%)	56 (86.2%)	35 (94.6%)	
Allo-HSCT	25 (52.1%)	40 (71.4%)	20 (57.1%)	0.11
Non-HSCT	23 (47.9%)	16 (28.6%)	15 (42.9%)	
Relapse	25 (46.3%)	18 (27.7%)	13 (35.1%)	0.11
Death	23 (42.6%)	11 (16.9%)	11 (29.7%)	0.009

Note: WBC: white blood cell; IQR: interquartile range; ACA: additional chromosomal abnormalities; Ph: Philadelphia chromosome; CNS: central nervous system; CR: complete remission; CRi: CR with incomplete hematologic recovery; EOI: end of induction; MRD: minimal residual disease; RT-qPCR: real-time quantitative polymerase chain reaction; Allo-HSCT: allogenic hematopoietic stem cell transplantation.

[*] Data are n (%).

[†] χ^2 or Fisher exact tests were performed to compare categorical variables between cohorts.

Supplemental Table 3. Baseline characteristics and treatment response of 156 adult patients based on *IKZF1*plus genotyping

	*IKZF1*plus (N = 54)*	non-*IKZF1*plus (N = 102)*	*P* value†
**Baseline characteristics**			
Gender			
Male	26 (48.1%)	58 (56.9%)	0.32
Female	28 (51.9%)	44 (43.1%)	
Age at diagnosis, years			
Median (range)	50 (20-75)	44.5 (19-76)	
18-39	14 (25.9%)	44 (43.1%)	0.09
40-64	34 (63.0%)	47 (46.1%)	
⩾ 65	6 (11.1%)	11 (10.8%)	
WBC count at diagnosis, x 10^9/L			
Median (IQR)	51.5 (30.9-130.2)	31.4 (10.7-82.7)	
< 30	13 (24.1%)	48 (47.1%)	0.01
⩾ 30	41 (75.9%)	54 (52.9%)	
Blasts count at diagnosis, %			
Median (IQR)	91.3 (85.0-94.4)	88.1 (73.4-94.0)	
< 90	20 (37.0%)	55 (53.9%)	0.06
⩾ 90	34 (63.0%)	47 (46.1%)	
CD19 expression ⩾ 20%			
Patients with available data	54 (100%)	99 (97.1%)	
Positive	54 (100%)	99 (100%)	1.00
Negative	0 (0%)	0 (0%)	
CD20 expression ⩾ 20%			
Patients with available data	54 (100%)	99 (97.1%)	
Positive	19 (35.2%)	5 (5.1%)	<0.001
Negative	35 (64.8%)	94 (94.9%)	
Cytogenetics at diagnosis			
Normal	9 (16.7%)	21 (20.6%)	0.92
Isolated Ph	12 (22.2%)	22 (21.6%)	
ACA	24 (44.4%)	45 (44.1%)	
Unknown	9 (16.7%)	14 (13.7%)	
CNS involvement			
Yes	2 (3.7%)	6 (5.9%)	0.71
No	52 (96.3%)	96 (94.1%)	
BCR::ABL1 transcript			
p190	44 (81.5%)	71 (69.6%)	0.26
p210	8 (14.8%)	21 (20.6%)	
p190 and p210	2 (3.7%)	10 (9.8%)	

**Treatment response**			
Tyrosine kinase inhibitor			
Imatinib	23 (42.6%)	44 (43.1%)	1.00
Flumatinib	31 (57.4%)	58 (56.9%)	
CR/CRi at EOI			
Yes	52 (96.3%)	97 (95.1%)	1.00
No	2 (3.7%)	5 (4.9%)	
MRD status at EOI, RT-qPCR			
Patients with available data	53 (98.1%)	98 (96.1%)	
MRD-	14 (26.4%)	20 (20.4%)	0.42
MRD+	39 (73.6%)	78 (79.6%)	
MRD status at 3 months, RT-qPCR			
Patients with available data	51 (94.4%)	90 (88.2%)	
MRD-	24 (47.1%)	38 (42.2%)	0.60
MRD+	27 (52.9%)	52 (57.8%)	
BCR::ABL1 mutation status			
Patients with available data	49 (90.7%)	91 (89.2%)	
Mutation	23 (46.9%)	37 (40.7%)	0.48
No mutation detected	26 (53.1%)	54 (59.3%)	
Transplantation			
Patients with available data	48 (88.9%)	91 (89.2%)	
Allo-HSCT	25 (52.1%)	60 (65.9%)	0.14
Non-HSCT	23 (47.9%)	31 (34.1%)	
Relapse	25 (46.3%)	31 (30.4%)	0.06
Death	23 (42.6%)	22 (21.6%)	0.009

Note: WBC: white blood cell; IQR: interquartile range; ACA: additional chromosomal abnormalities; Ph: Philadelphia chromosome; CNS: central nervous system; CR: complete remission; CRi: CR with incomplete hematologic recovery; EOI: end of induction; MRD: minimal residual disease; RT-qPCR: real-time quantitative polymerase chain reaction; Allo-HSCT: allogenic hematopoietic stem cell transplantation.

[*] Data are n (%).

[†] Fisher exact tests were performed to compare categorical variables between cohorts.

Supplemental Table 4. Internal validation of MLPA technique

MLPA P335 probes	Standard deviation (n = 8)	Standard deviation (n = 8)	MLPA P202 probes	Standard deviation (n = 16)
EBF1 - 16	0.02	0.03	ZPBP - 3	0.04
EBF1 - 14	0.02	0.05	IKZF1 - up	0.03
EBF1 - 10	0.02	0.02	IKZF1 - 1	0.04
EBF1 - 1	0.03	0.03	IKZF1 - 1	0.04
IKZF1 - 1	0.03	0.02	IKZF1 - 1	0.03
IKZF1 - 2	0.03	0.01	IKZF1 - Intr.1	0.03
IKZF1 - 3	0.03	0.03	IKZF1 - 2	0.03
IKZF1 - 4	0.03	0.04	IKZF1 - 2	0.02
IKZF1 - 5	0.03	0.06	IKZF1 - 3	0.04
IKZF1 - 6	0.02	0.05	IKZF1 - 3	0.02
IKZF1 - 7	0.02	0.02	IKZF1 - Intr.3	0.03
IKZF1 - 8	0.03	0.02	IKZF1 - Intr.3	0.02
JAK2 - 23	0.04	0.04	IKZF1 - 4	0.03
CDKN2A - 4	0.04	0.02	IKZF1 - 4	0.03
CDKN2A - 2	0.03	0.04	IKZF1 - 5	0.02
CDKN2B - 2	0.03	0.05	IKZF1 - 5	0.03
PAX5 - 10	0.01	0.04	IKZF1 - 6	0.02
PAX5 - 8	0.03	0.03	IKZF1 - 6	0.03
PAX5 - 7	0.04	0.03	IKZF1 - 7	0.02
PAX5 - 6	0.02	0.03	IKZF1 - 7	0.02
PAX5 - 5	0.02	0.03	IKZF1 - 8	0.03
PAX5 - 2	0.03	0.03	IKZF1 - 8	0.03
PAX5 - 1	0.02	0.04	FIGNL1 - 4	0.03
ETV6 - 1	0.03	0.03	CDKN2A - 4	0.03
ETV6 - 1	0.02	0.04	CDKN2A - 2	0.04
ETV6 - 2	0.02	0.03	CDKN2B - 1	0.03
ETV6 - 3	0.02	0.05	CEP170B - 10	0.04
ETV6 - 5	0.03	0.05	MTA1 - 14	0.04
ETV6 - 8	0.02	0.03	CRIP2 - 1	0.05
BTG1 - AREA - down	0.03	0.03	IGHD - up	0.04
BTG1 - AREA - down	0.02	0.04	KCNJ6 - 4	0.03
BTG1 - 2	0.03	0.04	ERG - 12	0.03
BTG1 - 1	0.03	0.03	ERG - 11	0.03
RB1 - 6	0.03	0.04	ERG - 10	0.01
RB1 - 14	0.03	0.01	ERG - 9	0.02
RB1 - 19	0.02	0.02	ERG - 8	0.03
RB1 - 24	0.02	0.04	ERG - 7	0.01

RB1 - 26	0.02	0.04	ERG - 6	0.03
SHOX - AREA - down	0.04	0.05	ERG - 5	0.05
CRLF2 - 4	0.04	0.06	ERG - 4	0.02
CSF2RA - 10	0.03	0.06	ERG - int 3	0.02
IL3RA - 1	0.03	0.02	ERG - 3	0.02
P2RY8 - 2	0.03	0.04	ERG - 2	0.02
ZFY - 4 (YC)	0.03	0.07	ERG - 1	0.03
COL11A1	0.01	0.04	ETS2 - 3	0.03
PEX13	0.03	0.03	COL11A1	0.03
SCN1A	0.02	0.03	DYSF	0.02
BRK1	0.03	0.03	EDAR	0.02
GBE1	0.05	0.06	SCN5A	0.03
CPOX	0.03	0.06	GBE1	0.03
SEMA5A	0.02	0.02	CTNND2	0.02
POR	0.02	0.03	ADGRV1	0.02
VPS13B	0.01	0.02	SLC6A5	0.04
COL5A1	0.04	0.02	MYBPC3	0.02
BEST1	0.02	0.03	COL2A1	0.04
FBN1	0.05	0.03	FBN1	0.02
ABAT	0.02	0.04	PLCG2	0.02
			CACNA1A	0.04
			SAMHD1	0.02

Note: For internal validation, 16 DNA samples from normal bone marrow of male patients had been used as normal genotype control. The results demonstrated that standard deviation for all probes in SALSA MLPA P335-C2 and P202-C1 were all ≤ 0.10, which totally met the criteria listed in the instructions for MLPA.

Supplemental Table 5. Baseline characteristics and treatment response of 128 patients stratified into three risk groups

	low-risk (N=37)*	intermediate-risk (N=43)*	high-risk (N=48)*	*P* value†
**Baseline characteristics**				
Gender				
Male	24 (64.9%)	25 (58.1%)	24 (50.0%)	0.38
Female	13 (35.1%)	18 (41.9%)	24 (50.0%)	
Age at diagnosis, years				
Median (range)	47 (20-64)	37 (19-63)	49 (20-64)	
18-39	13 (35.1%)	25 (58.1%)	14 (29.2%)	0.01
40-64	24 (64.9%)	18 (41.9%)	34 (70.8%)	
WBC count at diagnosis, x10^9/L				
Median (IQR)	21.2 (8.7-43.0)	38.7 (11.0-181.4)	52.2 (33.9-122.7)	
< 30	24 (64.9%)	17 (39.5%)	10 (20.8%)	<0.001
≥ 30	13 (35.1%)	26 (60.5%)	38 (79.2%)	
Blasts count at diagnosis, %				
Median (IQR)	87.4 (70.0-94.5)	90.0 (79.8-94.1)	91.0 (85.0-94.3)	
< 90	19 (51.4%)	21 (48.8%)	18 (37.5%)	0.38
≥ 90	18 (48.6%)	22 (51.2%)	30 (62.5%)	
CD19 expression ≥ 20%				
Patients with available data	34 (91.9%)	43 (100%)	48 (100%)	
Positive	34 (100%)	43 (100%)	48 (100%)	1.00
Negative	0 (0%)	0 (0%)	0 (0%)	
CD20 expression ≥ 20%				
Patients with available data	34 (91.9%)	43 (100%)	48 (100%)	
Positive	1 (2.9%)	2 (4.7%)	19 (39.6%)	<0.001
Negative	33 (97.1%)	41 (95.3%)	29 (60.4%)	
Cytogenetics at diagnosis				
Normal	13 (35.1%)	5 (11.6%)	9 (18.8%)	0.21
Isolated Ph	8 (21.6%)	8 (18.6%)	11 (22.9%)	
ACA	13 (35.1%)	22 (51.2%)	21 (43.8%)	
Unknown	3 (8.1%)	8 (18.6%)	7 (14.6%)	
CNS involvement				
Yes	1 (2.7%)	5 (11.6%)	2 (4.2%)	0.24
No	36 (97.3%)	38 (88.4%)	46 (95.8%)	
BCR::ABL1 transcript				
p190	30 (81.1%)	26 (60.5%)	39 (81.3%)	0.12
p210	6 (16.2%)	12 (27.9%)	8 (16.7%)	
p190 and p210	1 (2.7%)	5 (11.6%)	1 (2.1%)	

**Treatment response**				
Tyrosine kinase inhibitor				
Imatinib	17 (45.9%)	19 (44.2%)	22 (45.8%)	0.98
Flumatinib	20 (54.1%)	24 (55.8%)	26 (54.2%)	
CR/CRi at EOI				
Yes	37 (100%)	38 (88.4%)	46 (95.8%)	0.08
No	0 (0%)	5 (11.6%)	2 (4.2%)	
MRD at EOI, RT-qPCR				
Patients with available data	35 (94.6%)	43 (100%)	47 (97.9%)	
MRD-	20 (57.1%)	0 (0%)	11 (23.4%)	<0.001
MRD+	15 (42.9%)	43 (100%)	36 (76.6%)	
MRD at 3 months, RT-qPCR				
Patients with available data	37 (100%)	43 (100%)	45 (93.8%)	
MRD-	37 (100%)	0 (0%)	20 (44.4%)	<0.001
MRD+	0 (0%)	43 (100%)	25 (55.6%)	
BCR::ABL1 mutation status				
Patients with available data	35 (94.6%)	36 (83.7%)	43 (89.6%)	
Mutation	9 (25.7%)	19 (52.8%)	21 (48.8%)	0.04
No mutation detected	26 (74.3%)	17 (47.2%)	22 (51.2%)	
Transplantation				
Patients with available data	37 (100%)	43 (100%)	48 (100%)	
Allo-HSCT	23 (62.2%)	31 (72.1%)	25 (52.1%)	0.15
Non-HSCT	14 (37.8%)	12 (27.9%)	23 (47.9%)	
Relapse	5 (13.5%)	17 (39.5%)	25 (52.1%)	0.001
Death	5 (13.5%)	12 (27.9%)	23 (47.9%)	0.003

Note: WBC: white blood cell; IQR: interquartile range; ACA: additional chromosomal abnormalities; Ph: Philadelphia chromosome; CNS: central nervous system; CR: complete remission; CRi: CR with incomplete hematologic recovery; EOI: end of induction; MRD: minimal residual disease; RT-qPCR: real-time quantitative polymerase chain reaction; Allo-HSCT: allogenic hematopoietic stem cell transplantation.

[*] Data are n (%).

[†] χ^2 or Fisher exact tests were performed to compare categorical variables between cohorts.

Supplemental Table 6. Gene signatures of *IKZF1*^plus genotype in *BCR::ABL1*+ ALL

	logFC	adj.P.Val	Regulation	Bastian et al.*	Kim et al.#
KSR2	2.33	6.85E-08	upregulated	reported	
LPAR5	1.66	7.75E-08	upregulated	reported	
AEBP1	1.98	7.75E-08	upregulated	reported	
COL6A3	3.45	7.75E-08	upregulated	reported	reported
GRID2IP	1.93	2.85E-07	upregulated	reported	
KCNMB3	1.25	3.62E-07	upregulated		
SEMA6C	1.65	3.88E-07	upregulated	reported	
TUNAR	2.92	4.14E-07	upregulated		
CD9	2.09	4.99E-07	upregulated	reported	
BVES	2.15	5.19E-07	upregulated	reported	
GPR68	1.33	1.47E-06	upregulated		
ATRNL1	3.05	1.84E-06	upregulated	reported	reported
MS4A1	2.28	2.76E-06	upregulated	reported	
C14orf132	2.67	3.50E-06	upregulated	reported	
CCDC81	1.64	3.97E-06	upregulated		
KCNJ16	1.67	4.50E-06	upregulated	reported	
HPS4	1.18	5.76E-06	upregulated	reported	
CAPSL	1.81	5.77E-06	upregulated	reported	
GCHFR	1.59	5.94E-06	upregulated		
LRRC14B	2.21	6.47E-06	upregulated	reported	
NCF1	1.04	1.02E-05	upregulated	reported	
GJC1	1.7	1.06E-05	upregulated	reported	
CARMIL3	1.38	1.19E-05	upregulated		
TAFA1	1.9	1.19E-05	upregulated		
TCL1A	2.94	1.25E-05	upregulated	reported	reported
ERVH48-1	1.49	1.47E-05	upregulated		
RAB3B	1.64	2.02E-05	upregulated	reported	
CD24	1.22	2.28E-05	upregulated		
DBNDD1	1.65	2.30E-05	upregulated	reported	
TUBB3	1.6	2.37E-05	upregulated		
CYYR1	-3.77	7.75E-08	downregulated	reported	reported
MLLT3	-2.32	7.75E-08	downregulated	reported	reported
EGF	-2.19	7.75E-08	downregulated	reported	
FAM229B	-1.44	7.75E-08	downregulated	reported	
AKR1C3	-2.05	1.71E-07	downregulated	reported	reported
FOCAD	-1.39	3.02E-07	downregulated	reported	reported
MARCKS	-2.55	3.33E-07	downregulated		

PRSS57	-2.45	3.33E-07	downregulated	reported	
DOCK1	-3.33	2.05E-06	downregulated	reported	reported
MAP7	-1.14	2.90E-06	downregulated	reported	
MREG	-1.73	2.96E-06	downregulated	reported	
GALNT3	-1.66	3.58E-06	downregulated	reported	
GLIPR1	-1.09	4.37E-06	downregulated	reported	
CDKN2A	-2.56	4.50E-06	downregulated		
HPGDS	-1.65	5.94E-06	downregulated	reported	
TSPAN32	-1.52	6.25E-06	downregulated	reported	
CLIP4	-1.45	6.42E-06	downregulated	reported	reported
MN1	-2.23	6.87E-06	downregulated	reported	reported
KDM5B	-1.25	8.11E-06	downregulated	reported	
GATA2	-1.86	8.32E-06	downregulated	reported	
MYO1E	-1.73	1.06E-05	downregulated	reported	
SLC19A2	-1.46	1.06E-05	downregulated	reported	
PCBD1	-1.5	1.19E-05	downregulated	reported	
TBL1X	-1.04	1.51E-05	downregulated		
RTL8A	-1.63	1.99E-05	downregulated		
DUSP5	-1.86	1.99E-05	downregulated	reported	
DNAJC1	-1.04	2.02E-05	downregulated	reported	
NABP1	-1.32	2.30E-05	downregulated	reported	
PWWP2B	-1.72	2.49E-05	downregulated	reported	
CHODL	-1.09	2.76E-05	downregulated		

*: differentially expressed genes between multilineage and lymphoid main cluster

#: marker genes of three molecular subtypes in *BCR::ABL1*+ lymphoblastic leukemia

Reference

1. Chalandon Y, Thomas X, Hayette S, Cayuela JM, Abbal C, Huguet F, et al. Randomized study of reduced-intensity chemotherapy combined with imatinib in adults with Ph-positive acute lymphoblastic leukemia. Blood. 2015;125(24):3711-9.
2. Ribera JM, Garcia O, Montesinos P, Brunet S, Abella E, Barrios M, et al. Treatment of young patients with Philadelphia chromosome-positive acute lymphoblastic leukaemia using increased dose of imatinib and deintensified chemotherapy before allogeneic stem cell transplantation. Br J Haematol. 2012;159(1):78-81.
3. Schwab CJ, Jones LR, Morrison H, Ryan SL, Yigittop H, Schouten JP, et al. Evaluation of multiplex ligation-dependent probe amplification as a method for the detection of copy number abnormalities in B-cell precursor acute lymphoblastic leukemia. Genes Chromosomes Cancer. 2010;49(12):1104-13.
4. Stanulla M, Dagdan E, Zaliova M, Möricke A, Palmi C, Cazzaniga G, et al. IKZF1plus Defines a New Minimal Residual Disease–Dependent Very-Poor Prognostic Profile in Pediatric B-Cell Precursor Acute Lymphoblastic Leukemia. Journal of Clinical Oncology. 2018;36(12):1240-9.
5. Garcia-Aznar JM, Alonso S, Iglesias DU, de Ugarriza PL, Lopez CA, Balbin M, et al. Mapping the genetic features of T-ALL cases through simplified NGS approach. Clin Immunol. 2022;245:109151.
6. Fedullo AL, Messina M, Elia L, Piciocchi A, Gianfelici V, Lauretti A, et al. Prognostic implications of additional genomic lesions in adult Philadelphia chromosome-positive acute lymphoblastic leukemia. Haematologica. 2019;104(2):312-8.
7. Weng XQ, Shen Y, Sheng Y, Chen B, Wang JH, Li JM, et al. Prognostic significance of monitoring leukemia-associated immunophenotypes by eight-color flow cytometry in adult B-acute lymphoblastic leukemia. Blood Cancer J. 2013;3(8):e133.
8. Jin P, Jin Q, Wang X, Zhao M, Dong F, Jiang G, et al. Large-Scale In Vitro and In Vivo CRISPR-Cas9 Knockout Screens Identify a 16-Gene Fitness Score for Improved Risk Assessment in Acute Myeloid Leukemia. Clin Cancer Res. 2022;28(18):4033-44.
9. Dai YT, Zhang F, Fang H, Li JF, Lu G, Jiang L, et al. Transcriptome-wide subtyping of pediatric and adult T cell acute lymphoblastic leukemia in an international study of 707 cases. Proc Natl Acad Sci U S A. 2022;119(15):e2120787119.
